# Unmasking the Hidden Culprit: Capsule Endoscopy Reveals Diffuse Hookworm Infestation as a Cause of Obscure Gastrointestinal Bleeding and Refractory Anemia—A Case Report

**DOI:** 10.1002/ccr3.73108

**Published:** 2026-07-06

**Authors:** Samiyya Rasool Abbasi, Momina Mazhar Ali, Ramsha Habibullah Mangrio, Marrium Sultan Dar, Syeda Dua Azhar, Khabab Abbasher Hussien Mohamed Ahmed

**Affiliations:** ^1^ Jinnah Postgraduate Medical Centre Karachi Pakistan; ^2^ Faculty of Medicine University of Khartoum Khartoum Sudan

**Keywords:** anemia, capsule endoscopy, gastrointestinal hemorrhage, hookworm infections

## Abstract

A 70‐year‐old woman with transfusion‐dependent anemia and ongoing gastrointestinal bleeding had inconclusive findings on imaging, upper endoscopy, and colonoscopy. Capsule endoscopy revealed the diagnosis: diffuse hookworm infestation causing active bleeding throughout the small bowel and colon. Targeted anthelmintic therapy resolved her anemia completely.

## Key Clinical Message

When standard tests failed to explain a 70‐year‐old woman's persistent bleeding and severe anemia, capsule endoscopy uncovered the surprising culprit: a diffuse hookworm infestation. This case reminds clinicians to consider parasitic infections in refractory anemia and to use capsule endoscopy when conventional workup comes up empty.

## 1 Introduction

Iron deficiency is one of the most common nutrition problems worldwide. It can cause serious health issues, such as poor mental and physical development in babies, reduced ability to work, higher chances of early delivery, and in severe cases, even death of the mother or baby. Iron deficiency usually happens due to one or more of these three reasons: the body needs more iron than usual, not getting enough iron from food, or losing too much blood. In many cases, iron deficiency is caused by gastrointestinal diseases, which lead to blood loss or poor absorption of iron [1].

Gastrointestinal (GI) bleeding is a frequent medical issue that doctors often see in both hospital and clinic patients. There are three key terms used to describe GI bleeding: overt, occult, and obscure GI bleeding. Overt GI bleeding refers to bleeding that is visible to the eye, such as bright red blood or dark, digested blood found in vomit or stool. In contrast, occult GI bleeding is not visibly apparent but is detected through a positive fecal occult blood test or the presence of iron deficiency anemia [2]. When a patient presents with occult bleeding, it is recommended to begin the evaluation with esophagogastroduodenoscopy (EGD) and colonoscopy to identify a potential source. If these tests do not reveal a cause and the patient continues to show signs of GI blood loss—such as ongoing iron deficiency, positive stool tests, or any later visible bleeding—the condition is classified as obscure GI bleeding [2, 3].

Iron deficiency anemia predominantly affects young children and women in less‐developed countries. In these regions, the condition arises from multiple factors, including inadequate dietary iron intake, high fertility rates, and iron loss through feces due to parasitic infections like hookworm, schistosomiasis, and trichuriasis. Infectious diseases, especially parasitic infections, can cause iron loss from the body and also trigger inflammation‐related anemia, which reduces the availability of iron for the body's tissues. Hookworm infection ranks among the most significant parasitic diseases in humans based on Disability Adjusted Life Years (DALYs) lost. The chronic intestinal blood loss it causes can lead to iron‐deficiency anemia, contributing to long‐term morbidity. This blood loss is primarily due to the parasite's release of coagulases, which disrupt normal clotting and result in continuous bleeding into the stool, rather than direct blood consumption by the parasite [4].

Current diagnostic techniques for parasitic infections often lack adequate specificity and sensitivity. There is no single “gold standard” test available that can detect intestinal parasites with 100% accuracy. Therefore, this case report highlights the diagnostic challenges in a 70‐year‐old female presenting with unexplained anemia and to demonstrate the utility of capsule endoscopy (CE) as an effective diagnostic tool in identifying the underlying cause.

## 2 Case History/Examination Presentation

A 70‐year‐old female patient, with all spontaneous vaginal deliveries, without any known comorbidities, was admitted to Medicine Ward 23 from the Outpatient Department. She presented with shortness of breath (SOB), bloody stools, easy fatigability, and abdominal pain over the past 6 days. The SOB had a sudden onset, was progressive, and classified as New York Heart Association (NYHA) class III, accompanied by dizziness. It was not accompanied by chest pain or pedal edema. The SOB coincided with episodes of loose, bloody stools (3–4 times per day) from the onset, with a foul odor, difficult to flush, and without any identified triggers or alleviating factors.

## 3 Differential Diagnosis, Investigations, and Treatment

The patient also experienced significant easy fatigability, becoming dependent on her family for daily activities and spending most of her time bedridden. Additionally, she reported generalized, dull, non‐radiating abdominal pain with mild severity and no particular triggers or relief. She had received 14 packed cell volume (PCV) transfusions over 2 months. Initial lab results showed normocytic anemia with a hemoglobin (Hb) level of 5.3 g/dL, which increased to 8.2 g/dL following a transfusion of 3 pints of PCV. However, her Hb dropped again to 6.7 g/dL 4 days later.

Urine and stool detailed report (DR) examinations were unremarkable. An ultrasound of the whole abdomen revealed a hypoechoic liver, cholelithiasis, and a right renal corticomedullary cyst, but no specific cause of the bloody stools. A computed tomography (CT) abdomen with a GI bleed protocol angiography showed mild hepatomegaly and bilateral non‐obstructing renal calculi, with no identifiable source of bleeding. EGD and colonoscopy were performed to further evaluate the source of GI bleeding. The colonoscopy showed altered blood from the rectum to the cecum but was otherwise unremarkable. EGD revealed only mild pangastric erythema.

## 4 Conclusion and Results (Outcome and Follow‐Up)

**FIGURE 1:**
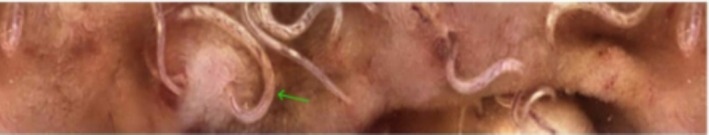
Small‐bowel capsule endoscopy image demonstrating multiple hookworms attached to the small‐intestinal mucosa, with associated luminal blood.

Due to inconclusive findings from prior tests, capsule endoscopy (CE) was conducted (Figure 1), revealing diffuse bleeding associated with multiple hookworms (morphologically consistent with hookworm species) throughout the duodenum, proximal/mid jejunum, and various sections of the colon. Melena‐like contents were also observed. Treatment comprised the anthelmintic mebendazole (100 mg orally twice daily for 3 days), together with supportive measures: tranexamic acid (500 mg intravenously [IV] as required), sucralfate suspension (2 tablespoons orally three times daily), co‐trimoxazole (sulfamethoxazole/trimethoprim, double‐strength 800/160 mg orally twice daily), and omeprazole (40 mg IV once daily). The patient's hemoglobin levels returned to normal with treatment and remained stable afterwards.

## 5 Discussion

This case highlights a 70‐year‐old female presenting with symptoms of GI bleeding, including shortness of breath, bloody stools, easy fatigability, and abdominal pain. Despite extensive investigations, including laboratory tests, imaging, colonoscopy, and EGD, the source of bleeding remained undetected. However, capsule endoscopy successfully identified diffuse GI bleeding associated with multiple hookworms throughout the duodenum, jejunum, and colon. Treatment with anthelmintic therapy and supportive care resulted in stabilization of the patient's hemoglobin levels, demonstrating the critical role of capsule endoscopy in diagnosing obscure GI bleeding when conventional methods are inconclusive.

Similar to our case, a case report by Walter et al. describes an elderly patient presenting with anemia where conventional diagnostic methods, including upper and lower endoscopy, failed to identify a clear source of bleeding. Capsule endoscopy proved essential in detecting the bleeding site within the small intestine, specifically the middle jejunal region, and even identified a foreign body as a potential contributing factor [5]. In our case, capsule endoscopy successfully identified multiple hookworms as the cause of diffuse GI bleeding, emphasizing its utility in detecting obscure sources of bleeding within the small intestine when standard diagnostic approaches are inconclusive. The comparison highlights the effectiveness of capsule endoscopy in uncovering elusive causes of GI bleeding, whether structural abnormalities or parasitic infections.

Hookworm infection, caused by the nematodes *Ancylostoma duodenale* and *Necator americanus* , is one of the most significant soil‐transmitted helminthic infections worldwide and a leading parasitic cause of iron‐deficiency anemia, particularly in developing countries [6, 7]. Transmission occurs chiefly through penetration of intact skin by filariform larvae present in faecally contaminated soil and, in the case of *A. duodenale*, through ingestion of larvae; the larvae subsequently migrate to the lungs and are swallowed, reaching the small intestine, where the adult worms attach to the mucosa of the duodenum and proximal jejunum [8, 9]. There, the worms feed on host blood and, through mucosal laceration and the secretion of anticoagulant and hydrolytic substances, produce chronic occult intestinal blood loss; in heavy or long‐standing infection this manifests as iron‐deficiency anemia and, less commonly, as overt gastrointestinal bleeding, as observed in our patient [9, 10]. Other clinical features may include abdominal pain, diarrhea, fatigue, and the constitutional symptoms of severe anemia [11]. According to the World Health Organization (WHO), soil‐transmitted helminths account for approximately 5.18 million DALYs globally, with hookworm responsible for the largest proportion of this burden [7, 12].

The mainstay of treatment for hookworm infection is anthelmintic therapy with a benzimidazole, which acts by binding parasitic β‐tubulin and impairing the worm's glucose uptake. The two most widely used agents are albendazole and mebendazole [13]. For hookworm specifically, single‐dose oral albendazole (400 mg) is generally preferred, as it achieves substantially higher cure rates than single‐dose mebendazole; in a randomized controlled trial, single doses of albendazole and mebendazole cured 69% and 29% of hookworm infections, respectively, whereas 3‐day regimens improved cure rates to 92% and 54% [13, 14]. Mebendazole—the agent administered to our patient—is therefore most effective against hookworm when given as a multi‐day course (100 mg twice daily for 3 days) rather than as a single dose [14]. Pyrantel pamoate is a recognized alternative where benzimidazoles are unavailable or contraindicated. Equally important is correction of the resulting anemia: oral or, where indicated, intravenous iron supplementation should accompany anthelmintic therapy, and transfusion of packed red cells is reserved for severe, symptomatic anemia, as was required in our patient. In co‐endemic settings, co‐administration of albendazole with praziquantel has been associated with a greater hemoglobin response [10]. Because reinfection and reduced benzimidazole efficacy are increasingly recognized concerns, alternative and combination agents—including tribendimidine, oxantel pamoate, and emodepside—are under active investigation, while at the population level the World Health Organization recommends preventive chemotherapy through mass drug administration alongside improvements in water, sanitation, and hygiene [11, 13].

Detecting intestinal parasites accurately remains challenging, as no single “gold standard” test exists with 100% sensitivity. The wet mount method in single stool examination has limited diagnostic yield due to its poor sensitivity [15]. While EGD is the standard diagnostic tool for gastric lesions, CE has gained attention as a valuable alternative. Unlike EGD, which can be uncomfortable for patients and carries risks of adverse events, particularly in high‐risk individuals when performed under sedation, CE offers a safer and less invasive option. By swallowing a small capsule equipped with a camera, CE captures detailed images of the entire gastrointestinal mucosa through natural peristaltic movements, providing a comprehensive and patient‐friendly diagnostic approach [16].

Although hookworm‐related obscure gastrointestinal bleeding is uncommon, it is increasingly recognized in endemic regions through small‐bowel capsule endoscopy. In a capsule endoscopy study from an endemic area, hookworm infestation was found to be a not‐uncommon cause of obscure occult and overt gastrointestinal bleeding [17]. Likewise, several reports have identified blood‐feeding hookworms attached to the proximal small‐bowel mucosa as the source of otherwise unexplained bleeding and refractory anemia, with prompt clinical and hematological recovery following anthelmintic therapy [18]. Our case adds to this growing literature and reinforces the value of capsule endoscopy in unmasking parasitic causes of obscure gastrointestinal bleeding, particularly in patients from, or with links to, hookworm‐endemic regions.

## 6 Conclusion

Numerous studies have assessed the diagnostic accuracy of CE compared to EGD, with highly promising results. Patients better tolerate CE, and its portable devices enable diagnostic evaluation even in remote areas with limited access to specialized healthcare facilities. These features make CE a valuable alternative to conventional EGD. Given the advances in technology and miniaturization of electronics, more sophisticated and miniaturized capsule endoscopy systems can be expected in the coming years.

## Author Contributions

**Samiyya Rasool Abbasi:** conceptualization, investigation, funding acquisition, writing – original draft, methodology, validation, visualization, writing – review and editing, software, formal analysis, project administration, data curation, resources, supervision. **Ramsha Habibullah Mangrio:** conceptualization, investigation, writing – original draft, writing – review and editing, visualization, validation, formal analysis, project administration, resources. **Marrium Sultan Dar:** writing – original draft, conceptualization, writing – review and editing, visualization, validation, supervision, software. **Momina Mazhar Ali:** conceptualization, investigation, funding acquisition, writing – original draft, methodology, validation, visualization, writing – review and editing, software, formal analysis, project administration, resources. **Syeda Dua Azhar:** writing – original draft, funding acquisition, conceptualization, writing – review and editing, visualization, validation, project administration, resources. **Khabab Abbasher Hussien Mohamed Ahmed:** writing – original draft, conceptualization, writing – review and editing, visualization, validation, supervision, project administration, software, methodology.

## Funding

The authors have nothing to report.

## Ethics Statement

The authors have nothing to report.

## Consent

Written informed consent was obtained from the patient to publish this report in accordance with the journal's patient consent policy.

## Conflicts of Interest

The authors declare no conflicts of interest.

## Data Availability

All the data that support the findings of this study are available from the corresponding author upon reasonable request.
